# Knockdown of OCT4 suppresses the growth and invasion of pancreatic cancer cells through inhibition of the AKT pathway

**DOI:** 10.3892/mmr.2014.2367

**Published:** 2014-07-07

**Authors:** HAI LIN, LI-HUA SUN, WEI HAN, TIE-YING HE, XIN-JIAN XU, KUN CHENG, CHENG GENG, LI-DAN SU, HAO WEN, XI-YAN WANG, QI-LONG CHEN

**Affiliations:** 1 Department of Pancreatic Surgery, The First Affiliated Hospital of Xinjiang Medical University, Urumqi, Xinjiang 830054, P.R. China; 2 Liver Disease Center, The First Affiliated Hospital of Xinjiang Medical University, Urumqi, Xinjiang 830054, P.R. China; 3 Department of Pancreatic Surgery, Tumor Hospital of Xinjiang Medical University, Urumqi, Xinjiang 830000, P.R. China

**Keywords:** octamer-binding transcription factor 4, pancreatic cancer, AKT, growth, invasion

## Abstract

Octamer-binding transcription factor 4 (OCT4) is one of the factors associated with self-renewal and differentiation in cancer stem cells, and is crucial for the progression of various types of human malignancy. However, the expression and function of OCT4 in human pancreatic cancer has not been fully elucidated. The purpose of the present study was to investigate the function and molecular mechanisms of OCT4 in pancreatic cancer cells. The clinical significance of OCT4 expression was assessed by an immunohistochemical assay using a tissue microarray procedure in pancreatic cancer tissues and cells with different degrees of differentiation. A loss-of-function approach was used to examine the effects of a lentivirus-mediated OCT4 small hairpin RNA vector on biological behaviors, including cell proliferative activity and invasive potential. The results demonstrated that the expression levels of OCT4 protein in cancer tissues were significantly elevated compared with those in adjacent non-cancerous tissues (65.0 vs. 42.5%; P=0.005), which was correlated with tumor differentiation (P=0.008). The knockdown of OCT4 inhibited the proliferation and invasion of pancreatic cancer cells (Panc-1) expressing high levels of OCT4, accompanied with decreased expression of AKT, proliferating cell nuclear antigen (PCNA) and matrix metalloproteinase-2 (MMP-2). In conclusion, the present study reveals that the increased expression of OCT4 is correlated with the differentiation of pancreatic cancer, while knockdown of OCT4 suppresses the growth and invasion of pancreatic cancer cells through inhibition of AKT pathway-mediated PCNA and MMP-2 expression, suggesting that OCT4 might serve as a potential therapeutic target for the treatment of pancreatic cancer.

## Introduction

Pancreatic cancer is recognized as the fourth most frequent cause of cancer-associated mortality, with an overall five-year survival rate of <1–2% (1). In China, pancreatic cancer is the sixth leading cause of mortality from malignant disease, with an overall cumulative five-year survival rate of 1–3% (2). Pancreatic cancer is usually not detected or diagnosed at the early stages of the disease, as there are no specific symptoms. An improved understanding of the molecular basis of host-tumor interactions may lead to significant progress in the development of new therapeutic agents and new therapeutic approaches (3).

Growing evidence has demonstrated that the aberrant expression of pluripotent stem cell-associated genes may confer primitive and aggressive traits and be associated with unfavorable clinical outcomes in certain types of solid cancer (4). Among these, octamer-binding transcription factor 4 (OCT4), a key transcription factor required to maintain the self-renewal and pluripotency of embryonic stem cells, has been identified to enhance the tumorigenesis of cancer stem cells (CSCs) (5) and malignant transformation of breast cells (6,7). An increased expression of OCT4 is associated with low differentiation, tumor, nodes and metastasis (TNM) staging and tumor recurrence in certain types of cancer, and serves as a promising biomarker for the diagnosis and prognosis of cancer patients (8–10). In addition, OCT4 is highly expressed in CSCs and is closely associated with resistance to chemotherapy (11,12). OCT4-expressing cancer cells show increased tumorigenicity and high resistance to chemotherapeutics (13). The expression of OCT4 is upregulated in neuroblastoma; however, it is inhibited by chemotherapy (14), suggesting that it may be a new target for identifying candidate antitumor drugs. Therefore, OCT4 may be important in carcinogenesis and may provide one possible mechanism by which cancer cells acquire a drug-resistant phenotype (15).

However, certain studies have demonstrated that Oct4 is not expressed in tumor cells that arise in autochthonous cancer models (16). Further investigation is required to understand the role and molecular mechanisms of OCT4 in cancer. In the present study, the expression of OCT4 was assessed by a immunohistochemical (IHC) assay using a tissue microarray procedure in cancer tissues and detected in pancreatic cancer cells with different degrees of differentiation. A loss-of-function approach was used to examine the effects of OCT4 on the biological behaviors of tumor cells. It was hypothesized that the expression of OCT4 may be correlated with the differentiation of pancreatic cancer, and knockdown of OCT4 suppressed certain biological behaviors of pancreatic cancer cells through inhibition of the AKT pathway.

## Materials and methods

### Materials

The pancreatic cancer cell lines (Bxpc3, Panc-1 and Mia PaCa-2) used for the experiments were obtained from the Institute of Biochemistry and Cell Biology (Shanghai, China). Human pancreatic cancer tissues were obtained from the Resource Sample Library of Major Disease of the First Affiliated Hospital of Xinjiang Medical University (Urumqi, Xinjiang, China). The lentivirus-mediated OCT4 small hairpin (sh) RNA vector (Lv-shOCT4), negative control vector (NC) and virion-packaging elements were purchased from GeneChem (Shanghai, China). OCT4 and AKT primers were synthesized by Applied Biosystems (Foster City, CA, USA). The tissue microarray of human pancreatic cancer was purchased from Shanghai Outdo Biotech Co., Ltd. (Shanghai, China). All antibodies were obtained from Santa Cruz Biotechnology, Inc. (Santa Cruz, CA, USA).

### Drugs and reagents

Dulbecco’s Modified Eagle’s medium (DMEM) and fetal bovine serum (FBS) were purchased from Thermo Fisher Scientific, Inc. (Waltham, MA, USA). TRIzol reagent and Lipofectamine 2000 were obtained from Invitrogen Life Technologies (Carlsbad, CA, USA). Moloney murine leukemia virus (M-MLV) reverse transcriptase was purchased from Promega Corporation (Madison, WI, USA). SYBR Green Master mix was obtained from Takara Bio, Inc. (Otsu, Japan) and the Enhanced Chemoluminscence (ECL) Plus kit was obtained from GE Healthcare (Piscataway, NJ, USA).

### Clinical samples and data

A tissue microarray was prepared for the immunohistochemical (IHC) test using a total of 40 consecutive cases of human pancreatic cancer tissues and corresponding adjacent non-cancerous tissues (ANCT), which were collected from the Department of Pancreatic Surgery between September 2005 and December 2011. The present study was approved by the Medical Ethics Committee of Xinjiang Medical University and written informed consent was obtained from the patients or their parents prior to sample collection. All the cases were reviewed by two pathologists and the clinical and histopathological data of the patients are summarized in Table I.

### Tissue microarrays

For each case, the tumor foci for construction of the tissue microarrays during routine diagnosis were selected by marking them on the hematoxylin and eosin-stained slide using a waterproof pencil. The Advanced Tissue Arrayer (ATA-100; Chemicon International, Tamecula, CA, USA) was used to create holes in a ‘recipient’ paraffin block and to acquire cylindrical core tissue biopsies with a diameter of 1 mm from specific areas of the ‘donor’ block. The tissue core biopsies were transferred onto the recipient paraffin block at defined array positions. The resulting tissue microarrays contained tissue samples from 40 formalin-fixed, paraffin-embedded cancer specimens with known diagnosis and correlated benign tumor tissues from patients.

The block was incubated in an oven at 45°C for 20 min to allow complete embedding of the grafted tissue cylinders in the paraffin of the recipient block and then stored at 4°C until microtome sectioning.

### IHC staining

Anti-OCT4 antibody (Wuhan Boster Biological Engineering Co., Ltd., Wuhan, Hubei, China) was used for IHC detection of the expression of OCT4 protein in tissue microarrays. Tissue microarray sections were processed for IHC analysis of OCT4 protein as follows: Tissue microarrays were incubated with biotinylated antibodies and horseradish peroxidase (Santa Cruz Biotechnology, Inc.). Anti-OCT4 antibody was used at a dilution of 1:200. Endogenous peroxidase was inhibited by incubation with freshly prepared 3% hydrogen peroxide with 0.1% sodium azide. Non-specific staining was inhibited with 0.5% casein and 5% normal serum (Invitrogen Life Technologies). Staining was developed using diaminobenzidine substrate and sections were counterstained with hematoxylin (Invitrogen Life Technologies). Normal serum or phosphate-buffered saline (PBS; Wuhan Boster Biological Engineering Co., Ltd.) was used to replace anti-OCT4 antibody in the negative controls.

### Quantification of OCT4 protein expression

OCT4 expression was semiquantitatively estimated as the total OCT4 immunostaining score, which was calculated as the product of a proportion score and an intensity score. The proportion score reflected the fraction of positively stained cells (score 0, <5%; score 1, 5–10%; score 2, 10–50%; score 3, 50–75%; score 4, >75%). The intensity score represented the staining intensity (score 0, no staining signal; score 1, weak positive signal; score 2, moderate positive signal; score 3, strong positive signal). Finally, a total expression score was provided, ranging between 0 and 12. A score of 0 was regarded as negative, a score of 1–3 was regarded as +, a score of 4–6 was regarded as ++, a score of 7–9 was regarded as +++ and a score of 10–12 was regarded as ++++. Two observers estimated the total immunostaining score, independently and blindly. The total score reported was the average of two observers.

### Cell culture and transfection

Pancreatic cancer cells were cultured in DMEM medium supplemented with 10% heat-inactivated FBS, 100 U/ml of penicillin and 100 μg/ml of streptomycin. The cells in this medium were placed in a humidified atmosphere containing 5% CO_2_ at 37°C. OCT4 shRNA and negative control lentivirus were transfected into Panc-1 cells. The cells were subcultured at a 1:5 dilution in medium containing 300 μg/ml G418. Positive, stable transfectants were selected and expanded for further investigation. The Lv-shOCT4-infected clone, the negative control vector-infected cells and Panc-1 cells were termed Lv-shOCT4, NC and CON groups, respectively.

### Quantitative polymerase chain reaction (qPCR)

To quantitatively determine the mRNA expression levels of OCT4 and AKT in the Panc-1 cell line, 7300 Real-time PCR system (Applied Biosystems) was performed. Total RNA was extracted from each clone using TRIzol reagent according to the manufacturer’s instructions. Reverse transcription was performed using M-MLV and cDNA amplification was performed using the SYBR Green Master mix kit according to the manufacturer’s instructions. The OCT4 gene was amplified using a specific oligonucleotide primer and the human GAPDH gene was used as an endogenous control. PCR conditions were as follows: 94°C for 30 sec, 56°C for 30 sec and 72°C for 90 sec, for 30 cycles, and a final extension at 72°C for 5 min. β-actin was used as a loading control. PCR products were analyzed by electrophoresis using a 2% agarose gel containing 0.1 mg/ml ethidium bromide fluorescent quantitation PCR (ABI-7500; Applied Biosystems). Data were analyzed using the comparative Ct method (2^−ΔΔCt^). Three separate experiments were performed for each clone.

### Western blot analysis

Panc-1 cells were harvested and extracted using lysis buffer [Tris-HCl, sodium dodecyl sulfate (SDS), mercaptoethanol and glycerol]. The cell extracts were boiled for 5 min in loading buffer and then an equal amount of cell extract was separated using 15% SDS-PAGE. The separated protein bands were transferred onto polyvinylidene fluoride membranes, which were subsequently inhibited in 5% skimmed milk powder. Primary antibodies against OCT4, AKT, proliferating cell nuclear antigen (PCNA) and matrix metalloproteinase-2 (MMP-2) were diluted according to the manufacturer’s instructions and incubated overnight at 4°C. Subsequently, horseradish peroxidase-linked secondary antibodies were added at a dilution of 1:1,000 and incubated at room temperature for 2 h. The membranes were washed three times with PBS and the immunoreactive bands were visualized using the ECL Plus kit according to the manufacturer’s instructions. The relative protein levels in different cell lines were normalized to the concentration of GAPDH. Three separate experiments were performed for each clone.

### Cell proliferation assay

Cell proliferation was analyzed using the MTT assay. Briefly, cells infected with Lv-shOCT4 were incubated in 96-well-plates at a density of 1×10^5^ cells per well with DMEM supplemented with 10% FBS. The cells were treated with 20 μl of MTT for 0, 24, 48 and 72 h and subsequently incubated with 150 μl of dimethyl sulfoxide for 5 min. The color reaction was measured at 570 nm using an automated enzyme immunoassay analyzer (Bio-Rad, Hercules, CA, USA). The proliferation activity was calculated for each clone.

### Transwell invasion assay

Transwell filters were coated with Matrigel (3.9 μg/μl; 60–80 μl) on the upper surface of a polycarbonate membrane (diameter, 6.5 mm; pore size, 8 μm). Following incubation at 37°C for 30 min, the Matrigel solidified and served as the extracellular matrix for analysis of tumor cell invasion. The harvested cells (1×10^5^) in 100 μl of serum-free DMEM were added into the upper compartment of the chamber. A total of 200 μl of conditioned medium derived from NIH3T3 cells was used as a source of chemoattractant, which was placed in the bottom compartment of the chamber. Following 24 h of incubation at 37°C with 5% CO_2_, the medium was removed from the upper chamber. The non-invaded cells on the upper side of the chamber were scraped off with a cotton swab. The cells that had migrated from the Matrigel^®^ into the pores of the inserted filter were fixed with 100% methanol, stained with hematoxylin and then mounted and dried at 80°C for 30 min. The number of cells invading through the Matrigel^®^ was counted in three randomly selected visual fields from the central and peripheral portion of the filter using an inverted microscope (CX21BIM-SET6; Olympus, Tokyo, Japan; magnification, ×200). Each assay was repeated three times.

### Statistical analysis

SPSS 20.0 was used for statistical analyses. The Kruskal-Wallis H test, χ^2^ test and one-way analysis of variance (ANOVA) were employed to analyze the expression rate in all groups. The least-significant differences method of multiple comparisons was used when the probability for ANOVA was statistically significant. P<0.05 was considered to indicate a statistically significant difference.

## Results

### Expression of OCT4 in pancreatic cancer tissues and cells

**Figure 1:**
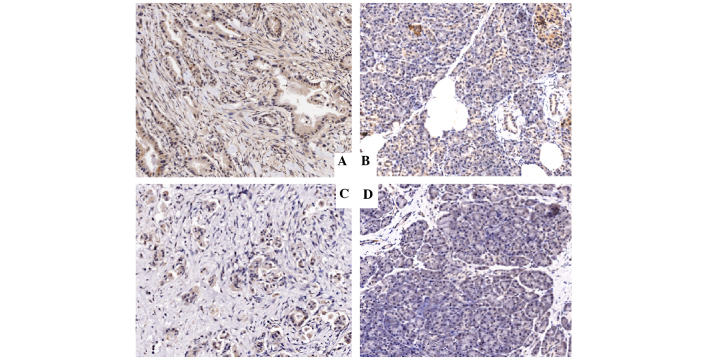
Expression of OCT4 protein in pancreatic cancer tissues (magnification, ×200). Pancreatic cancer tissues and ANCT were immunohistochemically stained with an anti-OCT4 antibody and classified as (−) and (+). (A) Positive expression in pancreatic cancer. (B) Negative expression in pancreatic cancer. (C) Positive expression in ANCT. (D) Negative expression in ANCT. Positive immunostaining of OCT4 was mainly localized in the nucleus of the tumor and ANCT cells. OCT4, octamer-binding transcription factor 4; ANCT, adjacent non-cancer tissues.

The expression of the OCT4 protein was assessed using IHC staining in pancreatic cancer tissues. As shown in Fig. 1, different levels of positive expression of the OCT4 protein were examined in pancreatic cancer tissues. Positive OCT4 immunostaining was mainly localized in the nucleus of cancer tissue cells. According to the OCT4 immunoreactive intensity, the positive expression of OCT4 in cancer tissues was significantly increased compared with that in ANCT (P=0.005; Table II).

**Figure 2:**
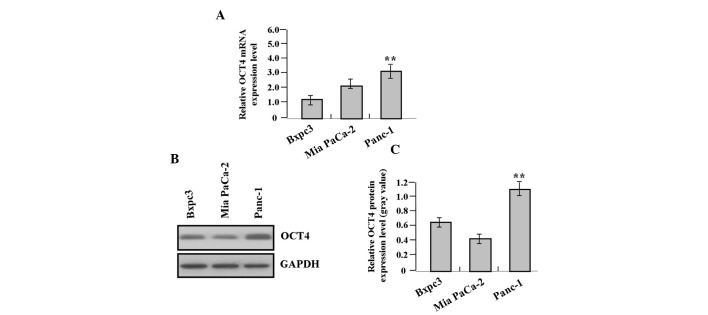
Expression of OCT4 in pancreatic cancer cells with different degrees of differentiation. The expression of OCT4 in pancreatic cancer cells with different degrees of differentiation (Bxpc3, Panc-1 and Mia PaCa-2) was examined by (A) real-time PCR and (B and C) western blot assays, of which OCT4 was highly expressed in the Panc-1 cell line compared with the other two cell lines (P<0.01). OCT4, octamer binding transcription factor 4.

The expression of OCT4 was detected in pancreatic cancer cells with different degrees of differentiation (Bxpc3, Panc-1 and Mia PaCa-2) by qPCR (Fig. 2A) and western blot analysis (Fig. 2B and C), of which OCT4 was highly expressed in the Panc-1 cell line compared with the other ones (P<0.01).

### Correlation of OCT4 expression with clinicopathological characteristics

The association between OCT4 expression and various clinical and histopathological features was analyzed. As shown in Table III, OCT4 expression was observed in 20/27 (74.1%) samples of the head of pancreatic cancer and 6/13 (46.2%) samples of the body and tail of pancreatic cancer. The increased expression of OCT4 protein was associated with the degree of differentiation in patients with cancer (P=0.008). However, no significant correlation was identified between OCT4 expression and lymph node metastases as well as age, gender, tumor sizes and sites in patients with pancreatic cancer (P>0.05).

### Effect of OCT4 knockdown on the expression of AKT

**Figure 3:**
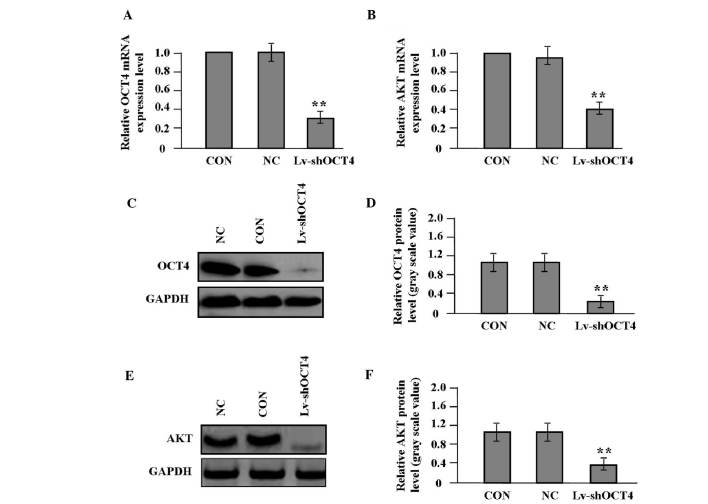
Effect of OCT4 knockdown on the expression of AKT in pancreatic cancer cells. After pancreatic cancer cells were transfected with the Lv-shOCT4 for 24 h, the expression levels of OCT4 and AKT were detected by (A and B) real-time PCR and (C–F) western blot analysis. The expression of OCT4 and AKT was significantly decreased in the Lv-shOCT4 group compared with the CON and NC groups (^**^P<0.01). OCT4, octamer binding transcription factor 4; Lv-shOCT4, lentivirus-mediated OCT4 shRNA vector; CON, control vector; NC, negative control vector.

After pancreatic cancer Panc-1 cells expressing a high level of OCT4 were stably transfected with Lv-shOCT4, the mRNA and protein expression levels of OCT4 and AKT were detected by qPCR (Fig. 3A and B) and western blot analysis (Fig. 3C–F). The results demonstrated that the expression of OCT4 and AKT was markedly decreased in the Lv-shOCT4 group compared with the NC and CON groups (P<0.01).

### Effect of OCT4 knockdown on cell proliferation

**Figure 4:**
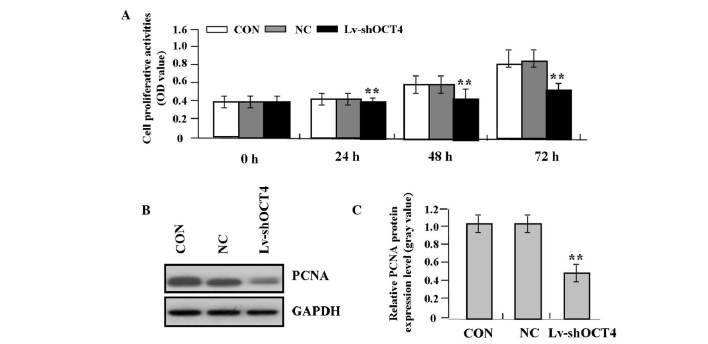
Effect of OCT4 knockdown on cell proliferation. (A) Cell proliferative activity, indicated by MTT assay, markedly decreased in a time-dependent manner in the Lv-shOCT4 group compared with the CON and NC groups (^**^P<0.01). (B and C) Endogenous expression of PCNA, indicated by western blot analysis, was significantly decreased in the Lv-shOCT4 group compared with the NC and CON groups (^**^P<0.01). OCT4, octamer binding transcription factor 4; Lv-shOCT4, lentivirus-mediated OCT4 shRNA vector; PCNA, proliferating cell nuclear antigen; CON, control vector; NC, negative control vector.

Deregulated cell proliferation is a hallmark of cancer. To investigate the effects of OCT4 knockdown on tumor growth in pancreatic cancer cells, the proliferative activities of Panc-1 cells were evaluated using the MTT assay. The present study found that OCT4 knockdown markedly decreased the proliferative activities of Panc-1 cells in a time-dependent manner compared with the NC and CON groups (Fig. 4A). In addition, the endogenous expression of PCNA, indicated by western blot analysis, was significantly decreased in the Lv-shOCT4 group compared with the NC and CON groups (P<0.01; Fig. 4B and C), indicating that knockdown of OCT4 may inhibit the invasive potential of pancreatic cancer cells through downregulation of PCNA expression.

### Effect of OCT4 knockdown on cell invasion

**Figure 5:**
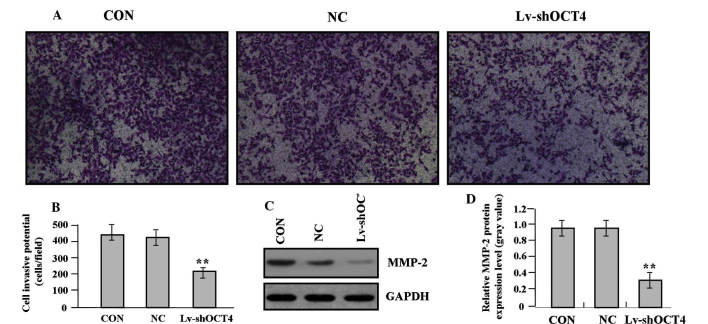
Effect of OCT4 knockdown on cell invasion (magnification, ×200). (A and B) Cell invasive potential, indicated by Transwell assay, was markedly weakened in the Lv-shOCT4 group compared with the CON and NC groups (^**^P<0.01). (C and D) Endogenous expression of MMP-2, indicated by western blot analysis, was significantly decreased in the Lv-shOCT4 group compared with the NC and CON groups (^**^P<0.01). OCT4, octamer binding transcription factor 4; Lv-shOCT4, lentivirus-mediated OCT4 shRNA vector; CON, control vector; NC, negative control vector; GAPDH, glyceraldehyde-3-phosphate dehydrogenase; MMP-2, matrix metalloproteinase-2.

To determine the effect of OCT4 knockdown on the invasive potential of pancreatic cancer cells, the Transwell assay was performed. The invasive potential of tumor cells in the Transwell assay was determined by the ability of cells to invade a matrix barrier containing laminin and type IV collagen, the major components of the basement membrane. Representative micrographs of Transwell filters are shown in Fig. 5A. It was revealed that the invasive potential of Panc-1 cells was apparently decreased in the Lv-shOCT4 group compared with the NC and CON groups (P<0.01; Fig. 5B). In addition, the endogenous expression of MMP-2, indicated by western blot analysis, was significantly decreased in the Lv-shOCT4 group compared with the NC and CON groups (P<0.01; Fig. 5C and D), indicating that knockdown of OCT4 may inhibit the invasive potential of pancreatic cancer cells through downregulation of MMP-2 expression.

## Discussion

CSCs are important in carcinogenesis and resistance to treatment, and may lead to metastasis. The isolation of circulating stem cells involves cell sorting based on the presence of cell surface markers, of which OCT4 has been reported to be overexpressed in colorectal cancer (CRC), including colitis-associated CRC (17–19). OCT4 has also been demonstrated to be associated with tumor growth and metastatic relapse (17,18). OCT4 positively regulates survivin expression to promote cancer cell proliferation and leads to a poor prognosis in esophageal squamous cell carcinoma (20,21). However, it has been demonstrated that OCT4B is decreased in prostate cancer and represents a strong biomarker of good prognosis for patients with prostate cancer (22). To elucidate the expression of OCT4 in cancer, its expression in human pancreatic cancer was assessed. It was revealed that the expression of OCT4 was elevated in the nucleus of cancer tissue cells and was associated with tumor differentiation; however, OCT4 did not correlate with tumor size and lymph node metastases. The present study, coupled with other studies, may indicate a possible association between OCT4 nuclear accumulation and turmorigenesis (23). OCT4 was also differentially expressed in pancreatic cancer cells with different degrees of differentiation, of which the Panc-1 cell line had the highest expression level of OCT4. Thus, the present study may provide a basis for further investigation of the function of OCT4 in pancreatic cancer with different degrees of differentiation.

In addition, OCT4 is more frequently located at the invasive front of tumors and correlates significantly with various aggressive behaviors and epithelial-mesenchymal transition (EMT) in nasopharyngeal carcinoma (24). The expression of OCT4 in melanoma cells increases the transmigration capacity, leading to high invasiveness and aggressiveness (25), and promotes cancer cell proliferation and colony formation (18,26). Inversely, knockdown of OCT4 inhibits CRC cell motility and invasion and decreases hepatic colonization (27), while patients with low Oct4 expression exhibit an improved overall survival rate (28). Similarly, the present study found that knockdown of OCT4 expression suppressed the proliferation and invasion of pancreatic cancer Panc-1 cells, suggesting that OCT4 may be an effective therapeutic target for the treatment of cancer.

Furthermore, certain studies have demonstrated that the AKT activation profile as well as its substrate spectrum are markedly correlated with the downregulation of OCT4 and are involved in the differentiation of embryonal carcinoma cells (ECC) (29). Reciprocal regulation of AKT and OCT4 promotes the self-renewal and survival of ECC (30). OCT4 post-translational modification-dependent interactions maintain restrained AKT signaling and promote a primitive epigenetic state (31). However, the present study found that the knockdown of OCT4 decreased the expression of AKT and suppressed the proliferation and invasion of pancreatic cancer cells with decreased expression of PCNA and MMP-2, while the expression of PCNA and MMP-2 is upregulated by AKT activation in pancreatic cancer cells (32). This suggests that OCT4 may be implicated in the development of pancreatic cancer through AKT pathway-mediated PCNA and MMP-2 expression.

In conclusion, the present study revealed that the increased expression of OCT4 is correlated with the degree of differentiation of pancreatic cancer, while knockdown of OCT4 suppresses the growth and invasion of pancreatic cancer cells through inhibition of AKT pathway-mediated PCNA and MMP-2 expression, suggesting that OCT4 may serve as a potential therapeutic target for the treatment of pancreatic cancer.

## Figures and Tables

**Table I:** Clinicopathological data of patients with pancreatic cancer.

Variable	No. of cases (%)
Patients, n	40 (100%)
Age, years	
<60	22 (55.0%)
≥60	18 (45.0%)
Gender	
Male	26 (65.0%)
Female	14 (35.0%)
Tumor size, cm	
<5	25 (62.5%)
≥5	15 (37.5%)
Tumor sites	
Pancreatic head	27 (67.5%)
Pancreatic body and tail	13 (32.5%)
Degree of differentiation	
High	11 (27.5%)
Moderate	20 (50.0%)
Low	9 (22.5%)
Distant metastases	
No	14 (35.0%)
Yes	26 (65.0%)

**Table II:** Expression of OCT4 protein in pancreatic cancer tissues.

Target	Variable	Case	Grading				Positive rate (%)	χ^2^	P-value

			−	+	++	+++			
OCT4	Pancreatic cancer	40	14	8	12	6	65.0		
	ANCT	40	23	12	4	1	42.5	7.927	0.005

OCT4, octamer-binding transcription factor 4; ANCT, adjacent non-cancerous tissues.

**Table III:** Correlation of OCT4 expression with the clinicopathological characteristics of patients with pancreatic cancer.

		OCT4 expression			
				
Variable	No. of cases	(−)	(+)	χ^2^	P-value
Total	40	14	26		
Age, years					
<60	22	7	15		
≥60	18	7	11	0.212	0.645
Gender					
Male	26	11	15		
Female	14	3	11	1.700	0.192
Tumor size, cm					
<5	25	8	17		
≥5	15	6	9	0.257	0.612
Tumor sites					
Pancreatic head	27	7	20		
Pancreatic body and tail	13	7	6	2.932	0.087
Degree of differentiation					
High	11	8	3		
Moderate	20	5	15		
Low	9	1	8	9.768	**0.008**
Lymph node metastases					
No	14	5	9		
Yes	26	9	17	0.005	0.945

OCT4, octamer binding transcription factor 4.
